# The Legacy of Infectious Disease Exposure on the Genomic Diversity of Indigenous Southern Mexicans

**DOI:** 10.1093/gbe/evad015

**Published:** 2023-02-02

**Authors:** Obed A Garcia, Kendall Arslanian, Daniel Whorf, Serena Thariath, Mark Shriver, Jun Z Li, Abigail W Bigham

**Affiliations:** Department of Anthropology, University of Michigan, Ann Arbor, Michigan; Department of Biomedical Data Science, Stanford University, Stanford, California; School of Public Health, Yale University, New Haven, Connecticut; College of Medicine, University of Illinois, Peoria, Illinois; Department of Anthropology, University of Tennessee, Knoxville, Tennessee; Department of Anthropology, Penn State University, State College, Pennsylvania; Department of Human Genetics, University of Michigan, Ann Arbor, Michigan; Department of Anthropology, University of California, Los Angeles, California

**Keywords:** natural selection, Mesoamerica, immune response, infectious disease, selection scan, evolutionary medicine

## Abstract

To characterize host risk factors for infectious disease in Mesoamerican populations, we interrogated 857,481 SNPs assayed using the Affymetrix 6.0 genotyping array for signatures of natural selection in immune response genes. We applied three statistical tests to identify signatures of natural selection: locus-specific branch length (LSBL), the cross-population extended haplotype homozygosity (XP-EHH), and the integrated haplotype score (iHS). Each of the haplotype tests (XP-EHH and iHS) were paired with LSBL and significance was determined at the 1% level. For the paired analyses, we identified 95 statistically significant windows for XP-EHH/LSBL and 63 statistically significant windows for iHS/LSBL. Among our top immune response loci, we found evidence of recent directional selection associated with the major histocompatibility complex (MHC) and the peroxisome proliferator-activated receptor gamma (PPAR-γ) signaling pathway. These findings illustrate that Mesoamerican populations' immunity has been shaped by exposure to infectious disease. As targets of selection, these variants are likely to encode phenotypes that manifest themselves physiologically and therefore may contribute to population-level variation in immune response. Our results shed light on past selective events influencing the host response to modern diseases, both pathogenic infection as well as autoimmune disorders.

**Significance**

Infectious diseases are known to shape immune response alleles worldwide, and population-specific variants that previously underwent natural selection could determine immunity to modern pathogens as well as the development of autoimmune disorders. Here, we identify a list of immune response genes in specific immune-related pathways that show extreme signatures of natural selection among an Indigenous Mesoamerican cohort. These genes are ideal candidates for future studies identifying host genetic factors influencing both susceptibility and resistance to modern pathogens. This study demonstrates the importance of natural selection in shaping the diversity of immune response alleles among Indigenous populations from Mexico.

## Introduction

Infectious diseases are among the strongest selective pressures acting on the human genome. Indeed, many genes subject to local positive natural selection (e.g., *CD40*) or balancing natural selection (e.g., *CCR5*, genes of the MHC complex) are associated with susceptibility to infectious disease (Hughes and Yeager 1998a; Bamshad et al. 2002; Sabeti et al. 2002a). Population genomic studies of global human populations show evidence of population-specific selection at immune response loci (Fan et al. 2016). Despite these advances, there remains a critical gap in our knowledge concerning selection at immune response loci among Indigenous Americans.

The diversity of infectious diseases in the Americas prior to colonial contact differed from the infectious diseases of Afro-Eurasia and Oceania. This was an outcome of both geographic isolation and differences in zoonotic biota (e.g., insects and fauna) that served as disease vectors. Therefore, selection likely did not act on the genomes of Indigenous Americans for variants that protected them from the Afro-Eurasian infectious diseases. Rather, pre-colonial populations in the Americas adapted to diseases that were locally prevalent, such as Chagas, tuberculosis, syphilis, and hepatitis (Merbs 1992; Klaus et al. 2010; Bos et al. 2014; Steverding 2014). Indigenous Americans' isolation from Afro-Eurasian infectious diseases ended with devastating effects. Beginning with European colonial contact in the late 15th century, there was a steady influx of novel infectious diseases to the Americas such as variola virus (smallpox) and measles virus—diseases for which indigenous communities across the Americas did not possess specific immunity. Across the Americas, famine, slavery, infectious disease, and warfare, contributed to the population collapse of various Indigenous American societies (Livi-Bacci 2006). Mitochondrial DNA data corroborate these historical accounts by demonstrating a population bottleneck 500 years ago coincident with European contact (O'Fallon and Fehren-Schmitz 2011). Accordingly, the evolutionary pressures for survival were strong. However, our knowledge of Indigenous American genetic variation in general and at loci related to infectious disease and immune response is limited. To date, only a few studies have identified genes under selection in Indigenous American populations (Eichstaedt et al. 2014; Lindo et al. 2016; Crawford et al. 2017; Mychaleckyj et al. 2017; Reynolds et al. 2019; Avila-Arcos et al. 2020). Among Mesoamericans, selection at immune response loci may have been particularly robust given this region's population density and level of urbanization throughout both pre- and post-colonial time periods (Smith 2005; Livi-Bacci 2006; Mummert et al. 2011). Furthermore, historical records from the colonial era indicate that novel infectious disease introduced by European colonizers (e.g., variola virus that causes smallpox) killed upwards of 90% of the indigenous communities in the region (Lockhart 1992; Feldman 1999; Restall et al. 2005; Leon-Portilla 2011). This high mortality rate led us to hypothesize that colonial contact left a strong signature of natural selection in the genomes of Mesoamericans at immune response loci.

Here, we interrogated SNP genotype data from Indigenous Mesoamericans for evidence of natural selection. We expected to identify a high proportion of immune response genes and pathways under natural selection given the history of infectious disease exposure among Mesoamericans across time.

## Results

### Mesoamerican Population Characteristics

Our Mesoamerican cohort included 39 individuals genotyped using the Affymetrix Genome-Wide Human SNP Array 6.0 containing 906,600 SNPs representing 25 Maya from the Yucatan Peninsula of Mexico, two Nahua, seven Mixtec, and five Tlapanec speakers from Guerrero, Mexico previously described in Bigham et al. (2010). Together, these individuals from different linguistic groups form a metapopulation that provides a shared history of selection in the population of Indigenous American particularly considering the much later diversification of languages than the dates calculated for the origin of the haplotypes under selection (Campbell 2000). Similar genetic data show that even though population substructure occurs among linguistic groups, the south forms a cluster with each other in Mesoamerica (García-Ortiz et al. 2021). We carried out statistical analysis using 857,481 autosomal SNPs that passed QC. We removed six individuals from the dataset that were first, second, or third-degree relatives, leaving us with a sample size of *n* = 33 individuals (supplementary table S1, Supplementary Material online; Manichaikul et al. 2010).

**Fig. 1.**
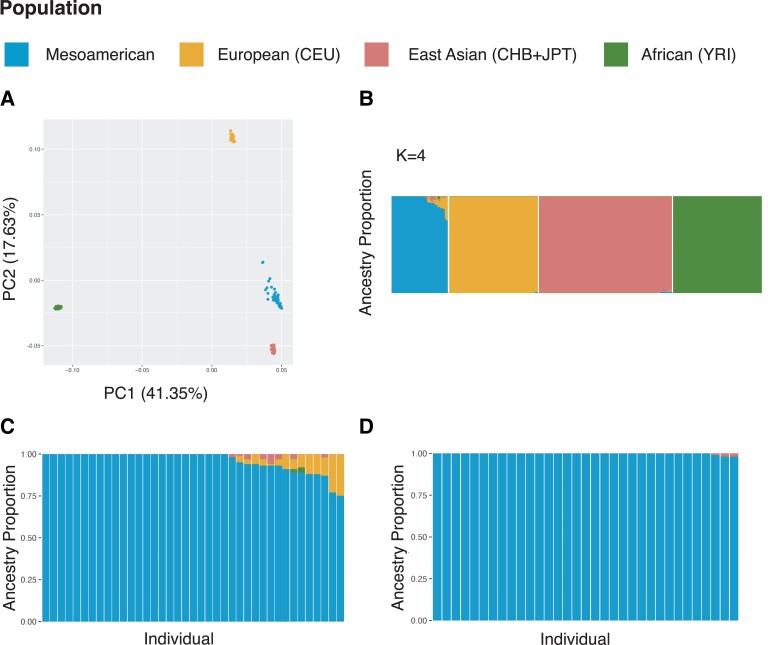
Individual ancestry estimates. Individual ancestry was estimated for Mesoamerican study participants using ADMIXTURE. (*A*) Principle component analysis for Mesoamericans, CEPH Europeans (CEU: Northern and Western Europeans from Utah), East Asians (CHB: Han Chinese from Beijing + JPT: Japanese from Tokyo), and Africans (YRI: Yoruba). PC1 explains 41.35% of the variance, while PC2 explains 17.63% of the variance observed. (*B*) ADMIXTURE global estimates (*K* = 4) of ancestry for Mesoamericans prior to masking admixture and removing related individuals. (*C*) All 39 Mesoamerican individuals prior to removing related individuals and correcting for admixture. (*D*) ADMIXTURE results for *N* = 33 and *K* = 4, after removing admixed segments using RFMIX2 and imputing missing genotypes using the unadmixed individuals for that specific chromosome from our cohort.

Indigenous Mesoamerican populations are known to exhibit varying degrees of European admixture (Bryc et al. 2010; Magalhaes et al. 2012). We performed a principal component analysis (PCA) in Plink 1.9, to visualize the relationship between our populations (fig. 1*A*; Purcell et al. 2007; Chang et al. 2015). In order to identify and remove the effects of European admixture from our selection scan, we estimated global ancestry using ADMIXTURE (Alexander and Lange 2011). We tested for four-way admixture including ancestry from the Americas, Europe, Africa, and East Asia (fig. 1*B*). Individual admixture estimates ranged from a maximum of 100% Indigenous American ancestry to a minimum of 75% Indigenous American ancestry, with most of our cohort possessing Indigenous Ancestry estimates above 90% (supplementary table S1, Supplementary Material online). European admixture was the most common of the three non-American ancestries (fig. 1*C*). It was detected in 15 individuals, ranging from 1% to 25%. Ten individuals had detectable East Asian ancestry ranging from 1% to 6%. African Ancestry was detected in two individuals at 3% and 2%.

Given the presence of non-Indigenous American ancestry within our final cohort of study participants, we assigned locus-specific ancestry to each chromosomal segment/haplotype using RFMix (Maples et al. 2013). To correct for admixture, which could be incorrectly detected as regions of selection, we set non-Indigenous American ancestry segments to missing and imputed the missing genotypes with SHAPEIT4 using the Indigenous American ancestry tracts from our dataset as the reference population (Delaneau et al. 2019). ADMIXTURE analysis performed on the masked and imputed Mesoamerican dataset indicated that this analysis effectively eliminated European and African ancestry from the Mesoamerican genomes (fig. 1*D*; supplementary tables S1 and S2, Supplementary Material online). After imputation, only three individuals had detectable East Asian ancestry less than 2%. Although recent scholarship such as Rodríguez-Rodríguez et al. (2022) has found substantial East Asian ancestry in Southern Mexico, we did not control for it as our IBD analysis also failed to detect any significant East Asian segments, therefore these are more likely due to shared ancestry rather than recent admixture events.

### Mesoamerican Genomes Show Evidence of a Population Bottleneck

**Fig. 2.**
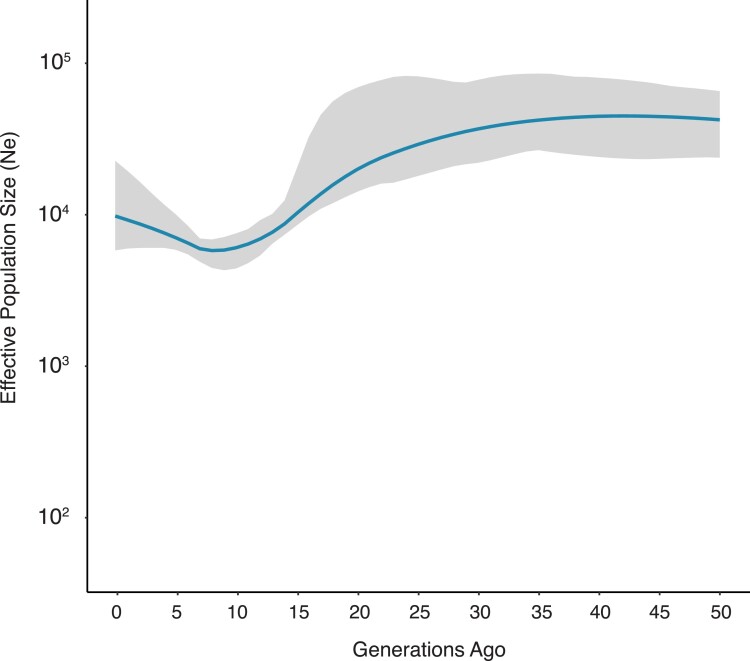
Effective population size estimates. Effective population sizes were calculated using AS-IBDNe. The *y*-axis represents the effective population size (*N*_e_). The *x*-axis represents the generation time. Mesoamericans experienced a bottleneck effect, with the lowest effective population size at eight generations ago (200 years ago, assuming a 25-generation time).

Mitochondrial DNA and historical records indicate that Indigenous American populations underwent a severe population bottleneck coincident with European contact beginning in the early 1500s (Lockhart 1992; Feldman 1999; Restall et al. 2005; Leon-Portilla 2011; O'Fallon and Fehren-Schmitz 2011). This bottleneck is hypothesized to be in large part caused by the introduction of novel infectious disease into the region. To detect evidence for this bottleneck, we estimated the historical effective population size of our cohort consisting of 33 Mesoamericans using the program AS-IBDne (Browning and Browning 2015; Browning et al. 2018). Our power is limited to reconstruct population effective size only to the first 50 generations as we used array data (Browning and Browning 2015). These data confirm that Mesoamericans went through a recent bottleneck, most likely associated with colonial contact (fig. 2). Fifty generations ago (∼1,250 years ago), Mesoamericans had a population size of roughly 86,400 people (95% bootstrap CI: 37,400−163,000). The vertex of the curve, or highest population effective size, was 42 generations ago with a population size of 94,100 (95% bootstrap CI: 36,700–21,0000). The data changes by a factor of 10 (from 10^4^ to 10^3^) between generations 15–16 (∼375–400 years ago). The base of the curve is evident at eight generations ago (∼200 years ago) with an effective population size of 4,800 (95% bootstrap CI: 3,270–6,130). While the confidence intervals are large throughout the dataset as a function of the small sample size analyzed, the effect of the bottleneck is noticeable with tighter 95% confidence intervals throughout the bottleneck (supplementary table S3, Supplementary Material online). This supports the bottleneck previously observed for individuals of Indigenous American ancestry (Browning et al. 2018; Mooney et al. 2018).

### Mesoamerican Genomes Show Evidence of Selection at Immune Response Loci

**Fig. 3.**
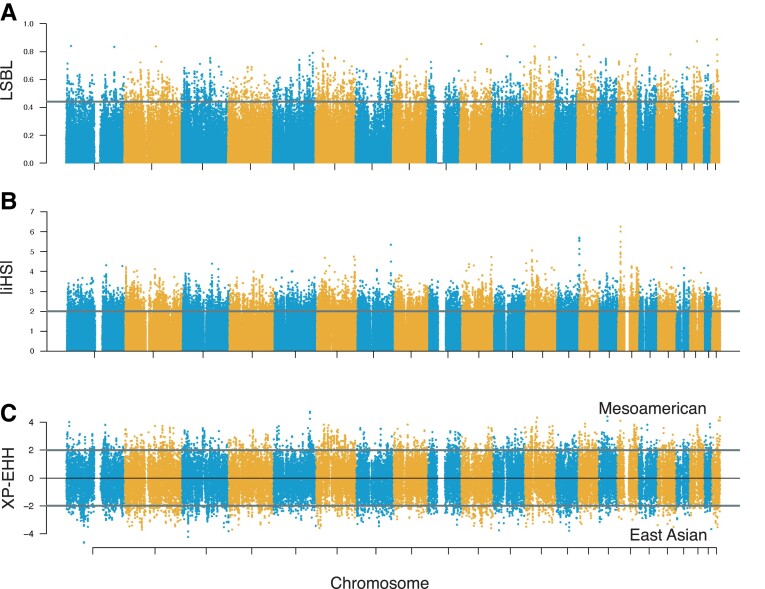
Manhattan plots of selection-scan statistics. For each plot, the value of the statistic is represented on the y-axis. Chromosome location is depicted along the x-axis. The thick horizontal lines indicate significance thresholds for each test statistic. (*A*) Distribution of LSBL values across the genome for Mesoamerican branch length calculated using East Asians and CEPH European Americans as outgroups. The line represents the 1% significance. (*B*) Plot of the absolute value of iHS scores for Mesoamericans. The line indicates scores of 2. The proportion of scores above 2 for each window is taken into consideration for determining the 1% significance. (*C*) Plot of XP-EHH comparing Mesoamericans to East Asians. Values above indicate directional selection in the Mesoamerican population whereas values below 0 indicate direction selection in East Asians. The line represents the values above or below 2, which Selscan flags as potentially significant above the 5% level.

To detect evidence of positive directional selection in Mesoamericans, we performed a selection scan using 33 Mesoamerican genomes whose non-indigenous chromosomal ancestry tracts were masked and imputed. We identified genomic signals of natural selection using three statistics: 1) locus-specific branch length (LSBL) (Shriver et al. 2004), 2) cross-population extended haplotype homozygosity (XP-EHH) (Sabeti et al. 2007; Pickrell et al. 2009), and 3) integrated haplotype score (iHS) (Voight et al. 2006). The EHH-based haplotype tests, XP-EHH and iHS, were calculated in Selscan (Szpiech and Hernandez 2014). In so doing, we leveraged both allele frequency difference and haplotype homozygosity to identify putatively selected regions of the genome. LSBL was calculated for each SNP in the dataset with a MAF ≥ 0.05 (497,699 SNPs) by comparing Mesoamericans to East Asians and Europeans. We identified 4,976 SNPs falling in the top 1% of the empirical distribution out of 497,699 total SNPs analyzed (fig. 3*A*). These SNPs exhibited Mesoamerican LSBL values from 0.442 to 0.887. The SNP with the most extreme LSBL value was *MRTFA* intronic variant rs17425081 located on chromosome 22. XP-EHH and iHS were calculated for non-overlapping windows of 100 kilobase pairs (kb). XP-EHH compared Mesoamericans to East Asians at 826,691 SNPs to look specifically for haplotypes present in Mesoamerican populations that arose after their split from Asian populations. iHS was calculated for 455,845 SNPs after filtering low-frequency variants. We identified 319 and 206 statistically significant 100 kb windows at the 1% level for XP-EHH and iHS, respectively (fig. 3*B*and*C*). These windows were scattered across the autosomes. Chromosome 6 contained the most significant windows of any chromosome for both XP-EHH and iHS, with 59 and 28 windows, respectively.

**Fig. 4.**
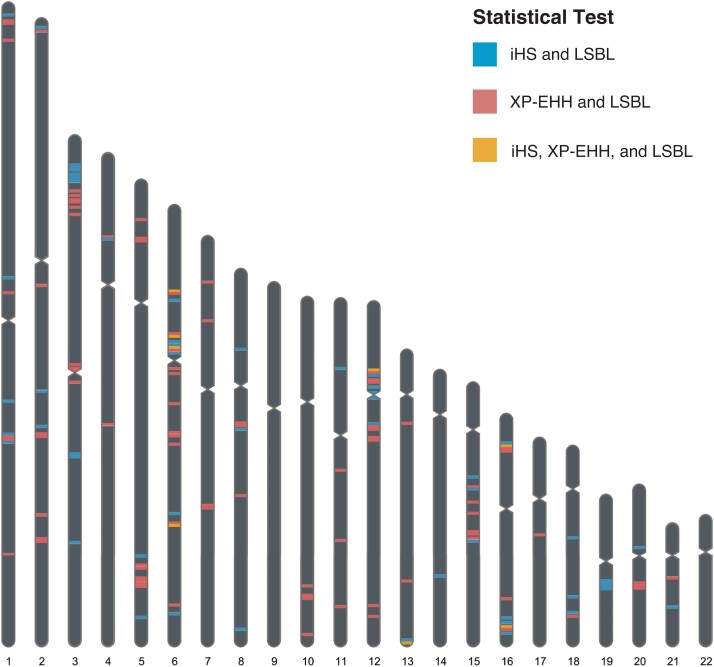
Genomic distribution of the 1% windows for iHS and XP-EHH when paired with LSBL. Regions that were in the 1% distribution for all three statistical tests are shaded in a lightest color. For both of the combined statistical tests, the majority of windows were found on chromosome 6, followed by chromosome 3.

To reduce false positives, we identified regions of the genome showing statistical significance for LSBL and at least one of the two haplotype tests, XP-EHH and iHS. To be considered significant, the XP-EHH and iHS windows were designated to be in the 1% tail by Selscan and that window needed at least one significant LSBL SNP also at the 1% level. Ninety-five significant regions at *P* < 0.01 were identified for the LSBL/XP-EHH analysis and 63 for the LSBL/iHS analysis (fig. 4). These regions were scattered across the genome and found on every autosome except chromosome 9 and chromosome 22. For the iHS/LSBL analysis, chromosomes 3 and 6 tied for the most significant regions of any chromosome, with 10 windows falling in the top 1% on each of the two chromosomes. Chromosome 6 contained the most significant regions for the XP-EHH/LSBL analysis with 15 windows, followed by chromosomes 3 and 12 with 10 each. Most of the significant results for chromosome 6 were identified in and around the major histocompatibility complex (MHC), a region essential for the adaptive immune response.

One of the largest contiguous regions of statistical significance was found on chromosome 3 (chr3:12,300,001–12,700,001). This 4 MB region consisted of four, tandem significant 100 kb windows containing the following genes: *PPARG*, *MKRN2*, *MKRN2OS*, *TSEN2*, and *RAF1*. Twenty-four of the 64 SNPs genotyped for this region fell in the top 1% of the empirical distribution for LSBL. Here, our most extreme LSBL value was 0.683 (rank 116) for the intronic SNP rs4684106 located in *TSEN2* followed by the *TSEN2* upstream variant rs17279604 (LSBL = 0.683, rank 117) and intronic variant rs17036821 (LSBL = 0.683, rank 118). There were several other extreme LSBL values including the *RAF1* non-coding transcript variant rs1051208 (LSBL = 0.640, rank 274), the *MKRN2OS* intronic variant rs17036922 (LSBL = 0.593, rank 671), and the *PPARG* intronic variant rs17793693 (LSBL = 0.581, rank 824). The most extreme iHS value was for the regulatory region variant to *PPARG*, rs9833097 (iHS = −3.73082). Furthermore, 11 of the 24 SNPs analyzed in this gene fell in the top 1% of the LSBL empirical distribution.

A second compelling result was the identification of two related regions on separate chromosomes. The first region was a 500 kb window located on chromosome 16 (chr16:11,000,001–11,500,001) containing the immune response genes *SOCS1* and *CIITA*, along with *DEXI*, *CELC16A*, *PRM1*, *PRM2*, *PRM3*, *TNP2*, *MIR548H2*, and *RMI2*. This region, particularly *CIITA*, is known to directly regulate MHC Class II expression, the second related region for which we found strong evidence of natural selection (Devaiah and Singer 2013; Sarmah et al. 2019). Within the first region, a 300 kb window (11,200,001–11,500,001) was significant for XP-EHH/LSBL and two 100 kb windows (11,000,001–11,100,001 and 11,200,001–11,300,001) were significant for iHS/LSBL. SNP rs4414511, located in the putative uncharacterized protein LOC400499, had the highest LSBL value (LSBL = 0.723, rank 49) for the region. Four additional extreme LSBL outliers, rs17605165, rs40448, rs28769, and rs193773, ranked 64, 68, 69, and 74, respectively, were identified in this region. Each of these four SNPs were in the lincRNA RP11-396B14.2, lying immediately upstream (∼30 kb) of *SOCS1*. The second related region was a 200 kb window located on chromosome 6 (chr6:33,000,001–33,200,001) containing the genes *HLA-DPA1*, *HLA-DPB1*, *HLA-DPB2 (pseudogene)*, *COL11A2*, *HCG24*, *HSD17B8*, *MIR219A1*, *RING1*, *RXRB*, and *SLC39A7*. This region was significant for XP-EHH/LSBL and included a nested 100 kb window (33,000,001–33,100,001) that was significant for iHS/LSBL. In fact, this region contained the highest number of significant LSBL/XP-EHH and LSBL/iHS windows of any region analyzed. Our highest LSBL value was for rs3128918 (LSBL = 0.752, rank 27), followed by rs3130578, rs3130179, rs3128952, and rs3130180 (ranked 98, 99, 201, and 202, respectively). Eight additional SNPs fell in the 1% XP-EHH/LSBL tail. To resolve which HLA alleles were part of the signature of selection, we imputed the classical HLA alleles for *HLA-DPA1* and *HLA-DPB1* using the multi-ethnic HLA reference panel in the Michigan Imputation Server (Das et al. 2016; Luo et al. 2021). *HLA-DPA1* was resolved to be HLA-DPA1*01:03 (AF = 0.93, rsq = 0.67) and *HLA-DPB1* was resolved to be HLA-DPB1*04:02 (AF = 0.89, rsq = 0.78). Given the high frequency in our dataset, these alleles form a single long-range haplotype, HLA-DPA1*01:03/DPB1*04:02. These allele frequencies and HLA haplotypes (HLA-DPA1*01:03/DPB1*04:02) were cross-referenced and concordant with previously reported HLA allele frequencies across Mexico, Central, and South America in the Allele Frequency Net Database (AFND) (Gonzalez-Galarza et al. 2019). To further confirm our imputed HLA allele frequencies and rule out unknown alternatives contributing to our imputation results, we accessed the publicly available high coverage whole genomes for individuals belonging to the Maya, Mixe, Mixtec, and Zapotec populations (*N* = 28) in the HGDP and SGDP projects (Mallick et al. 2016; Bergström et al. 2020). We used HLA-LA to call the DPA1 and DPB1 HLA alleles at the G-group resolution level (Dilthey et al. 2019). For DPA1, coverage ranged from 30.4 to 96.4, with an average of 45.5. For DPB1, the coverage ranged from 18.9 to 70.9, with an average of 43.9. In this new dataset, DPA1*01:03's frequency was 94.33% and DPB1*04:02's frequency was 74.47%. While our DPA1*01:03 frequency was similar between the two datasets, 93% versus 94.33%, our DPB1*04:02 frequency was higher in our dataset, 89% versus 74.47%. However, both DPB1*04:02 frequencies are high, giving us confidence in our imputation.

A third compelling result was a 200 kb window located on chromosome 5 (chr5:153,800,001–154,000,001) containing the genes *GALNT10*, *HAND1*, *MIR3141*, *SAP30L*, and *SAP30L-AS1*. For this window, 6 of the 18 SNPs tested for LSBL and all 54 of the SNPs tested for XP-EHH fell in the top 1% of the results. The intergenic SNP, rs4958377, exhibited the highest LSBL value, 0.580 (rank 829), followed by the non-coding transcript exon variant, rs2351485, located in lncRNA region CTB-158E9 (LSBL = 0.560, rank = 1,149). The most extreme XP-EHH value within the window was 4.67 for SNP rs880083. Of note, the haplotype “core” for XP-EHH may be present just outside of the window, where rs7710430 had the max XP-EHH score for that region (chr5:153,797,277, XP-EHH value = 4.761, LSBL = 0.556, rank 1,207).

For our combined LSBL-haplotype analysis, we identified several other significant chromosomal regions containing genes involved in immune response pathways that stood out given what is known about Mesoamerican population history. They included regions on chromosomes 2, 5, 6, 8, 12, and 15 that included the immune response genes *CHIA*, *IL18R1*, *IL18RAP*, *DOCK2*, *CYP7A1*, *IL17F*, *RPAP3*, *ENDOU*, and *TCF12* (table 1). Windows containing these genes displayed LSBL values ranging from 0.791 (rank = 10) for the *DOCK2* intronic variant, rs264838, to 0.449 for the *CHIA* intronic variant, rs1266828 (rank = 4,612). Five of these windows contained LSBL values falling in the top 200 and included the genes *DOCK2*, *TCF12*, *RPAP3/ENDOU*, and *CYP7A1*. Of note, upstream from the *RPAP3/ENDOU* window lies an extreme LSBL value for the regulatory region variant, rs2051827 (LSBL = 0.837, rank = 7). Six windows were significant for XP-EHH with values ranging from 2.43 for the regulatory region variant rs10201184 located in the window containing *IL18R1*/*IL18RAP* to 4.02 for *RPAP3*/*ENDOU* intergenic SNP rs667610. Two windows contained significant iHS scores including *DOCK2* (rs155239 = −3.70).

**Table 1:** Table of Immune Response Genes in the 1% of a Haplotype Test and LSBL

Gene	Chr	Window (hg19)	Hap. test	Max test imputed	Max test admixed	*N* unadmixed subset	Max test unadmixed	Max LSBL	Ohana LSBL	Max LSBL SNP ID
*IL18R1*, *IL18RAP*	2	103,000,001–103,100,001	XP-EHH	2.43	2.55	NA	NA	0.504	0.493	rs4851007
*PPARG*, *MRKN2*, *RAF1*	3	12,300,001–12,700,001	iHS	−3.73^a^	2.98	NA	NA	0.683	0.617	rs4684106
*GALNT10*	5	153,800,001–154,000,001	XP-EHH	4.76	4.83	NA	NA	0.58	0.518	rs4958377
*DOCK2*	5	169,100,001–169,200,001	iHS	−3.7	−3.45	NA	NA	0.791	0.717	rs264838
*HLA-DPA1*, *HLA-DPB1*, *HLA-DPB2*, *RING1*, *RXRB*	6	33,000,001–33,100,001	iHS and XP-EHH	−4.69 and 3.82	−4.41 and 2.56^b^	22	3.51 (XP-EHH)	0.752	0.576	rs3128918
*IL17F*	6	52,100,001–52,200,001	XP-EHH	2.89^a^	2.65^b^	25	3.36	0.486	0.401	rs1266828
*CYP7A1*	8	59,400,001–59,500,001	XP-EHH	3.83	3.49	NA	NA	0.746	0.669	rs12545426
*SOCS1*, *CIITA*	16	11,000,001–11,500,001	iHS and XP-EHH	−6.26^a^ and 3.01	−5.46 and 2.76	NA	NA	0.723	0.683	rs4414511
*ENDOU*	12	48,000,001–48,200,001	XP-EHH	4.1	4.16	NA	NA	0.765	0.685	rs10082722
*CHIA*	1	111,800,001–111,900,001	XP-EHH	3.02	3.06	NA	NA	0.449	0.352	rs17027272
*TCF12*	15	57,200,001–57,500,001	XP-EHH	2.78^a^	2.17^b^	27	2.69	0.705	0.618	rs2585110

Immune response genes are listed for each significant region. Genes not related to immunity within the window are not listed here.

For regions significant for both iHS and XP-EHH, the maximum test statistic for iHS is listed first followed by the maximum test statistic for XP-EHH.

a Indicates that the maximum test score is proximate to the statistical window and is most likely the haplotype core as detected by the selection scan test.

b Indicates that the window fell to a 5% of significance and warranted an extra set of analysis.

We performed gene ontology pathway enrichment analysis in DAVID (Huang et al. 2009a, 2009b) to identify overrepresented associations of genes and gene groups. We limited our analysis to the significant regions identified from the LSBL/XP-EHH or the LSBL/iHS analysis. DAVID analysis identified 21 biological processes gene ontology (GO) terms for the LSBL/XP-EHH analysis (supplementary table S4, Supplementary Material online) and 16 biological processes GO terms for the LSBL/iHS analysis (supplementary table S5, Supplementary Material online). We also performed a DAVID analysis using the combined set of significant genes for both LSBL/XP-EHH and LSBL/iHS, identifying 33 biological processes GO terms (supplementary table S6, Supplementary Material online). Several pathways related to immune function were identified including positive regulation of chemokine secretion, immune response, signal transduction, positive regulation of transcription from RNA polymerase II promoter, and positive regulation of interferon-gamma production. None of the fold enrichment *P*-values remained significant after correcting for multiple tests.

In addition to our DAVID analysis, we performed a Reactome pathway analysis in SNP-NEXUS (Chelala et al. 2009; Dayem Ullah et al. 2018; Jassal et al. 2019). Our iHS/LSBL hits falling in the 1% represent categories related to amino acid transport and transcriptional regulation (supplementary table S7, Supplementary Material online). For results in the 1% of the XP-EHH/LSBL analysis, our strongest signals were related to homeostasis, IL-18 signaling, TGF-β signaling, and pro-inflammatory response (supplementary table S7, Supplementary Material online). The combined XP-EHH/iHS/LSBL analysis introduced additional signal transduction categories (supplementary table S7, Supplementary Material online). Expanding the analysis to the 5% cutoff, we see overrepresentation of PPARG-related transcription factors, intracellular signaling by second messengers, and interferon-gamma signaling, among other immune-related categories (supplementary tables S8–S10, Supplementary Material online).

To determine whether imputing from our own dataset overly homogenized regions and biased our selection scan results, we generated admixture-corrected allele frequencies using the program Ohana with *K* = 4 to control for any European, East Asian, and African genetic contributions to Mesoamericans (Szpiech and Hernandez 2014; Cheng et al. 2022). Using the Ohana matrix results, we recalculated LSBL on the Mesoamerican admixture-corrected allele frequencies. Our significant results remained significant in this new analysis. For instance, *IL18R1* SNP rs4851007, had an LSBL value of 0.504 (*P*-value 0.0047) for our original calculations and 0.493 (*P*-value 0.0027) for the calculations using Ohana. Admixture adjusted LSBL values for our top candidate loci are reported in table 1, and they all remained in the 1% tail of LSBL values. We also tested for the effects of imputation on XP-EHH and iHS. To do this, we used the original dataset (with admixture and without imputation) to recalculate XP-EHH/iHS and normalized as described above. Eight of the 11 regions under selection remained in the 1% significance for the haplotype tests (table 1). Three regions that contained HLA-DPA1, HLA-DPB1, HLA-DPB2, RING1, RXRB, IL17F, and TCF12 dropped to the 5% significance level but only in the haplotype tests. For these regions, we re-estimated iHS and XP-EHH using only individuals with Indigenous American ancestry. We find that even though these regions fell to the 5% significance using the admixed individuals, using an unadmixed cohort brings the results back into the 1%. Therefore, our methods show that certain important immune loci could be missed when looking at an admixed cohort with a small sample size.

To gain insight into the age and spread of putatively selected genomic regions identified here, we generated haplotype age estimates for eleven haplotypes using extended haplotype homozygosity (EHH) scores based on the results of gene grouping related to immune response from the DAVID analysis. To do so, EHH scores were log-transformed and linearly regressed to the distance from the core SNP (Sabeti et al. 2002b; Voight et al. 2006; Szpiech and Hernandez 2014). The haplotypes showing evidence of selection range in age from roughly 4,000 to 10,000 years (table 2). This translates to 162–380 generations when assuming a 25-year generation time. Thus, their introduction predates colonial contact and has implications for natural selection operating on standing variation. For the HLA haplotype, HLA-DPA1*01:03/DPB1*04:02, we estimated that it arose 6,000 years ago, and then increased in frequency through natural selection in response to infectious diseases.

**Table 2:** Estimate of Haplotype Ages Using EHH

Gene	Chr	Window (hg19)	r2	Generations ago	Generations (95% CI)	Age (25 yr gen)	Age (95% CI)
*CHIA*	1	111,800,001–111,900,001	0.61	162.23	145.25–179.21	4,055.85	3,631.31–4,480.25
*IL18R1*, *IL18RAP*	2	103,000,001–103,100,001	0.87	335.29	322.24–348.34	8,382.20	8,056.00–8,708.50
*PPARG*, *MRKN2*, *RAF1*	3	12,300,001–12,700,001	0.86	320.46	298.43–342.49	8,011.50	7,460.71–8,562.27
*GALNT10*	5	153,800,001–154,000,001	0.93	192.58	187.92–197.25	4,814.55	4,697.97–4,931.14
*DOCK2*	5	169,100,001–169,200,001	0.90	212.81	205.39–220.24	5,320.35	5,134.83–5,505.90
*HLA*-*DPA1*, *HLA*-*DPB1*, *HLA*-*DPB2*, *RING1*, *RXRB*	6	33,000,001–33,100,001	0.89	240.13	231.13–249.13	6,003.28	5,778.34–6,228.23
*IL17F*	6	52,100,001–52,200,001	0.87	199.11	188.97–209.24	4,977.68	4,724.29–5,231.06
*CYP7A1*	8	59,400,001–59,500,001	0.97	362.09	353.06–371.11	9,052.28	8,826.50–9,278.00
*ENDOU*	12	48,000,001–48,200,001	0.65	264.20	233.38–295.01	6,605.00	5,834.56–7,375.24
*TCF12*	15	57,200,001–57,500,001	0.77	380.64	355.14–406.15	9,516.00	8,878.515–10,153.65
*SOCS1*, *CIITA*	16	11,000,001–11,500,001	0.91	186.63	176.85–196.41	4,665.78	4,421.33–4,910.23

## Discussion

The pathogenic history of the Americas has undoubtedly impacted the suite of genetic variation present among Indigenous Americans. Nonetheless, our knowledge of Indigenous American genetic variation at immune response loci is incomplete, leaving a critical gap in our understanding of the genomic consequences of infectious disease exposure in the Americas. We hypothesized that genetic variation at immune response loci was shaped by natural selection among Indigenous Americans during their unique history of infectious disease exposure, including exposure to pathogens prevalent in the Americas prior to European contact as well as newly introduced infectious diseases arriving during the contact period. We show that Mesoamerican populations experienced a sizeable population bottleneck coincident with the arrival of Europeans in the Americas. This lends support to the hypothesis that newly introduced infectious diseases shaped extant patterns of genomic variation. To this end, we find evidence of natural selection in regions of the genome involved in the body's immune response in support of this hypothesis. Together, our data highlight the importance of immunity and adaptation among Mesoamerican populations whether deep in our evolutionary past, as recent as colonial contact, or continuously shaped by recent infectious diseases.

Leveraging two orthogonal population genomic statistics that detected departures from neutrality, LSBL and XP-EHH/iHS, we identified signatures of natural selection in regions of the genome involved in the body's immune response. We identified 100 and 57 statistically significant windows for the LSBL/XP-EHH and LSBL/iHS analysis, respectively. Of these, three stood out as particularly compelling with respect to immune adaptation. The first was a 4 MB region located on chromosome 3 (chr3:12,300,001− 12,700,001) containing the gene *PPARG*, or *Peroxisome Proliferator-Activated Receptor Gamma*. It is a ligand-activated transcription factor that contributes to gene regulation as part of the PPAR-γ signaling pathway, which regulates lipid and glucose metabolism through the expression of cytokines and chemokines (Le Menn and Neels 2018). Importantly, the PPAR-γ signaling pathway activates both pro- and anti-inflammatory macrophages (Chawla 2010). The second was a region on chromosome 5 containing the gene *GALNT10*. *GALNT10* interacts with MHC complex genes as well as various interleukin cytokines (Kakoola et al. 2014) and is responsible for regulating CD4^+^ T cells infiltration of macrophages and decreasing granzyme B expression in CD8^+^ T cells (Zhang et al. 2020). CD4^+^ T cells are crucial to immune memory and CD8^+^ T cells are essential for protection against viruses, intracellular bacterial infection, and tumor cells (Worthington et al. 2012). It should be cautioned, given the continuous legacy of infectious disease exposure in Mesoamerica, any gene(s) in this region could have been the target of past selection.

The third compelling result included two related regions residing on separate chromosomes 6 and 16. The chromosome 6 result was anticipated given the presence of the MHC, a known region of high genomic diversity that contains 224 genes largely related to immunity (Trowsdale 1993; de Bakker et al. 2006). The MHC complex has been identified numerous times in natural selection scans performed in human populations and across other mammalian and aquatic species (Hughes and Yeager 1998b). We hypothesized that the haplotype, HLA-DPA1*01:03/DPB1*04:02, is most likely the target of selection given its primary role as a cell surface receptor in antigen-presenting cells—crucial to recognizing foreign pathogens. Hepatitis B (HB) may have driven selection on this haplotype across time given the continuous presence of pre- and post-colonial lineages of the virus. Both HLA-DPA1*01:03 and HLA-DPB1*04:02 alleles independently have been shown to be protective for HB infection and known to play a role in developing long-term seroprotective immunity following HB vaccination among East Asian populations (Chung et al. 2019; Ou et al. 2019, 2021; Wang et al. 2019, 2021; Sanchez-Mazas 2020). HB infection previously was thought to have originated in the Americas, but ancient DNA analysis has demonstrated that it most likely co-evolved with humans as we dispersed across the globe (Muhlemann et al. 2018). Therefore, lineages existing in the Americas and novel HB lineages introduced through European contact, in conjunction with shifting social demographics, likely shaped the HLA diversity among Indigenous American populations although uncertain to know for certain (Guzman-Solis et al. 2021). The second related region on chromosome 16 contained the genes, *class II, major histocompatibility complex transactivator* (*CIITA*), known to positively regulate chromosome 6 MHC Class II expression, and *suppressor of cytokine signaling 1* (*SOCS1*) (Reith et al. 2005; Krawczyk and Reith 2006; Devaiah and Singer 2013). *SOCS1* activation inhibits *CIITA* activation and therefore subsequent MHC Class II expression as part of the IFN-γ pathway (O'Keefe et al. 2001). We identified a cluster of SNPS exhibiting extreme LSBL values residing in the lincRNA, RP11-396B14.2. The gene targets of this lincRNA are currently unknown, but it lies immediately upstream of *SOCS1*. This provides evidence for natural selection acting on variation affecting transcription. Together, these two related windows on chromosomes 6 and 16 illustrate the potential importance of selection acting on complimentary regions.

Given the lack of publicly available data for larger cohorts of Indigenous American populations, we did not compare Mesoamerican genomes to other Indigenous American genomes to identify region-specific selective events. Therefore, our study design was unable to distinguish if a selective event was specific to Mesoamericans or affected Indigenous American populations more broadly. However, by comparing our results to other research identifying evidence of selection in the Americas, we were able to identify genes or chromosomal regions that overlap across studies or were distinct to our analysis. One particularly noteworthy gene with overlapping evidence of selection in our Mesoamerican cohort as well as among the Amerindian ancestry component of Brazilians from Mychaleckyj et al. (2017) was *CIITA*. In two complementary LSBL analyses performed by Mychaleckyj et al. (2017), one promoter region SNP, rs6498115, and one intronic SNP, rs45601437, from this gene where among the topmost differentiated SNPs. rs6498115 was included on the Affymetrix 6.0 array, whereas rs45601437 was not. In our analysis, rs6498115 was among four SNPs in the *CIITA* region that fell in the top 1% of the empirical distribution for LSBL. This overlap between our studies lends further support to the hypothesis that *CIITA* variation was the target of selection during the Asia to America migration or during the peopling of the Americas in a population ancestral to both Mesoamericans and Brazilians.

Genes in pathways that control the body's response to infectious disease, as well as to climate, altitude, and metabolic traits, show the strongest selection signatures in the human genome (Sabeti et al. 2007; Grossman et al. 2013). Notably, our immune system is highly redundant and compensates for factors such as novel genetic variation that may be detrimental to specific pathways. Furthermore, genetic markers regulating the immune response are general and diverse in function. For these reasons, evolutionary changes in allele frequencies brought about by natural selection to more ancient pathogens are likely to affect the pathogenesis of modern infectious diseases. For instance, *CIITA* continues to be important by providing resistance factors to modern infectious diseases such as Ebola virus and SARS-CoV-2 (Bruchez et al. 2020), and *IL18R1* has been shown to confer protection against more severe clinical dengue phenotypes through IL1α downregulation (Yeo et al. 2014). However, not all genomic variation is protective against modern infectious agents. Variants in *SOCS1* increase susceptibility to and disease progression of Influenza A and SARS-CoV-2 (Bhattacharjee and Banerjee 2020; Johnson et al. 2020; Lee et al. 2020). Therefore, we can leverage regions of the genome showing signatures of selection to identify resistance and/or susceptibility loci to modern pathogenic infection. This approach can be a particularly attractive strategy for studies with a limited-sized study population (Werren et al. 2021). In fact, focusing on genes under selection has proven beneficial in smaller sample sizes as demonstrated by several studies taking this approach (Park et al. 2012; Schwarzenbacher et al. 2012; Karlsson et al. 2013; Perry et al. 2014). The immune response genes identified here can provide an excellent starting point for genomic susceptibility studies of infectious diseases burdening modern Mesoamerican populations, while also providing greater statistical power to test fewer variants in smaller cohorts. Furthermore, they may be useful in studies seeking to understand cross-immunity between various infectious diseases of the period.

Similarly, targeting immune response genes subject to past natural selection can aid in the study of population-specific variants related to metabolic disease, autoimmune disease, or cancer given that many of the pathways are overlapping. For example, *PPARG* and *CYP7A1* regulate cholesterol homeostasis and metabolism, with documented effects of *CYP7A1* polymorphisms on statin metabolism across worldwide populations (Chinetti et al. 2001; Kajinami et al. 2004; Thompson et al. 2005; Baker et al. 2010; Wei et al. 2011; Li et al. 2013; Kadam et al. 2016). The IL-17 pathway, of which *IL17F* is a part of, is an important target for various autoimmune disorders (Hu et al. 2011), and a variant in *GALNT10* is highly associated with asthma susceptibility in a meta-analysis of populations of Latin American ancestry (Torgerson et al. 2011). Follow-up studies to our selection scan using highly differentiated alleles in populations of Mesoamerican ancestry would increase statistical power to identify associations with complex disease as exemplified by the study of Ko et al. (2014) on risk alleles involved in dyslipidemia.

There were several limitations to our study. First, our results are based on SNP microarray data, which inherently suffers from ascertainment bias. The Affymetrix 6.0 chip was designed to capture the diversity and haplotype structure of the HapMap Project populations, European Americans of northern and western European descent (CEU), East Asians (JPT and CHB), and Yorubans (YRI). Linkage disequilibrium blocks and SNP distribution is expected to differ in Mesoamerican populations. Therefore, our analysis may have failed to identify candidate genes and gene regions for natural selection, but it is unlikely to suffer from a high rate of false positivity. Additionally, this SNP microarray has been used successfully to identify signatures of natural selection in several human populations including Indigenous Americans, cementing its usefulness in population genomics studies of natural selection (Bigham et al. 2010). Second, the haplotype tests used in our analysis required ancestral allele information for each SNP. Any SNP without this information was removed from the XP-EHH and iHS analyses. As a result, several chromosomal windows contained insufficient SNP density for calculations of XP-EHH and iHS. Lastly, our analysis for effective population size was limited due to the number of individuals included in the analysis and the array ascertainment bias, leading to wider confidence intervals. However, a population bottleneck with much smaller confidence intervals was clearly visible, coinciding with the time period of colonial contact. A better designed SNP array or the interrogation of sequencing data would remedy these caveats in future studies.

## Conclusions

We present the results of a natural selection scan performed in Indigenous Mesoamerican populations from Mexico. We find evidence for a population bottleneck coincident with the arrival of Europeans to the Americas and natural selection in genes related to both adaptive and innate immunity. We suggest that past selective events influence the host response to modern diseases, both pathogenic infection as well as autoimmune disorders. Therefore, searching for signatures of past natural selection in genes related to immune function is a particularly attractive strategy for identifying host genetic factors influencing both susceptibility and resistance to disease. Together, our findings provide valuable insight into Mesoamerican population history and identify candidate loci for studying localized, biological responses to modern infectious and autoimmune disease.

## Methods

### Populations

Our Mesoamerican cohort included a total of 39 individuals representing the following populations: Twenty-five Maya from the Yucatan Peninsula of Mexico, two Nahua, seven Mixtec, and five Tlapanec speakers from Guerrero, Mexico previously described in Bigham et al. (2010). We obtained publicly available data from The 1000 Genomes Project Consortium for the following control populations: 60 Europeans of Northern and Western European ancestry (CEU), 90 East Asians from Beijing, China (CHB) and Tokyo, Japan (JPT), and 90 Yoruba from Ibadan, Nigeria (YRI) (International HapMap Project 2003; The 1000 Genomes Project Consortium 2012, 2015).

### Genome-wide SNP Data

All samples were previously genotyped using the Affymetrix Genome-Wide Human SNP Array 6.0 containing 906,600 SNPs (Bigham et al. 2010). We analyzed autosomal SNPs with call rates >95. The X and Y-chromosome as well as mitochondrial DNA (mtDNA) SNPs were excluded from our analyses as we chose to focus on the autosomes. No SNPs were removed based on departure from Hardy-Weinberg equilibrium (HWE) as this could potentially remove SNPs under selection that would mimic HWE departures. After QC, we carried out statistical analysis using 857,481 autosomal SNPs.

### Phasing and File Manipulation

All files were haplotype-phased using SHAPEIT4, processed using PLINK 1.9/2.0, and manipulated using VCFtools and BCFtools (Purcell et al. 2007; Li et al. 2009; Danecek et al. 2011; Chang et al. 2015; Delaneau et al. 2019). 1000 Genomes Project phase 3 populations were used for phasing the SNP data (The 1000 Genomes Project Consortium 2015).

### Relatedness

We calculated relatedness using kinship coefficients as estimated with the Kinship-based INference for Genome-wide association studies (KING) (Manichaikul et al. 2010). We removed six individuals from our dataset that were first, second, and third-degree relatives, leaving us with a sample size of *n* = 33 individuals.

### Admixture Analysis

PCA was conducted in PLINK 1.9 (Purcell et al. 2007; Chang et al. 2015). Global estimate of admixture for each Mesoamerican individual (*n* = 39) was calculated using an unsupervised model in ADMIXTURE (*K* = 4) (Alexander and Lange 2011). Chromosomal segment ancestry was estimated using RFMix2, assuming approximately 20 generations since initial admixture, which corresponds to admixture on Spanish encomiendas in the Yucatan (Machuca Gallegos 2016); (Maples et al. 2013). A 3-population admixture model between Indigenous Americans (SGDP), Africans (YRI), and Europeans (CEU) was assumed, as we believe the segments containing East Asian ancestry were due to shared ancestry and not a result of admixture. We used the Viterbi segment assignments to extract haplotypes demonstrating admixture, set genotypes in these regions as missing using bedtools, and imputed the missing segments on each chromosome with PBWT imputation in SHAPEIT4 using a customized reference panel (Quinlan and Hall 2010; Quinlan 2014; Delaneau et al. 2019). This customized reference panel was designed on a chromosome-by-chromosome basis comprised of our own unadmixed Mesoamerican individuals for that chromosome to impute the “missing” segments (supplementary table S2, Supplementary Material online). Given that the proportions of admixture were small, we imputed off our own dataset as the homogenization of haplotypes would be minimal.

### Ancestral Alleles

Ancestral alleles were queried from the 1000 Genomes Project phase 3 VCF files using BCFtools (Li et al. 2009). VCF files were recoded using PLINK 2.0 to preserve phasing information (Purcell et al. 2007; Chang et al. 2015). The dataset used for XP-EHH and iHS contained 841,217 SNPs, as only SNPs with ancestral allele information were used for haplotype testing.

### Estimating Historical Effective Population Size

To estimate effective population size in our cohort, and account for admixture, we used the Ancestry-specific Identity by Descent Effective Population size (AS-IBDne) (Browning et al. 2018). We used van Eeden et al. (2022)'s adapted snakemake AS-IBDne pipeline to calculate the Indigenous American effective population size in our 33 Mesoamericans (https://github.com/hennlab/AS-IBDNe). The primary deviation from the original AS-IBDne method is that this pipeline incorporates the local ancestry from RFMix2 instead of the RFMix 1.5.4 output. For references, we used 22 Indigenous Americans from the SGDP project, 22 Han Chinese, 22 European Americans, and 22 Yoruba from the HapMap project (International HapMap Project 2003; Mallick et al. 2016). Breaks and gaps in the IBD segments caused by phasing or genotype errors were filtered using the merge-ibd-segments program as part of the *Refined IBD* suite, setting no more than one discordant homozygote, and removing IBD segments shorter than 0.6 cM (Browning and Browning 2013). Historical effective population size and its 95% confidence were calculated using default parameters with a filter to analyze segments larger than 4 cM as appropriate for array data in *IBDne* (Browning and Browning 2015). A generation time of 25 years was assumed to transform the generations ago into years before present. Results were restricted to 50 generations before present as IBDne underestimates effective population size for SNP array data (supplementary table S3, Supplementary Material online; Browning and Browning 2015). After QC and filtering our IBD results, we only yielded AS-IBDne results for Indigenous American, European, and African ancestry. No putative East Asian segments passed our filters. As we are primarily interested in the effective population size of our Mesoamerican cohort, we only used the output corresponding to Indigenous American ancestry.

### Selection Scan

We employed three statistics to identify regions in the genome showing statistical evidence of natural selection: 1) LSBL (Shriver et al. 2004), 2) XP-EHH (Sabeti et al. 2007; Pickrell et al. 2009), and 3) iHS (Voight et al. 2006). LSBL compared Mesoamericans against European Americans and East Asians. We filtered the dataset for SNPs with an MAF > 0.05, which left us with 497,699 SNPs to analyze for LSBL. *F*_st_ values were computed for each SNP using Weir-Cockerham's equation (Weir and Cockerham 1984; Shriver et al. 2004; Akey 2009; Bigham et al. 2010). Statistical significance was determined using an empirical distribution. *P*_E_(*x*) = (number of loci > *x*)/(total number loci) using a significance threshold of α = 0.01 (Akey et al. 2002). LSBL results were then aggregated into 100 kilobase pair windows, that matched with the XP-EHH and iHS coordinates.

XP-EHH and iHS were calculated in Selscan (Szpiech and Hernandez 2014). XP-EHH was calculated for 826,691 autosomal SNPs, whereas iHS was calculated for 455,845 autosomal SNPs after filtering out low-frequency variants as this statistic was not designed to capture low-frequency variant information or alleles near fixation. XP-EHH was genome-wide normalized using the norm function. iHS was standardized based on allele frequency bins, normalizing the SNPs in quantiles organized by similar frequencies, again using the norm function. We grouped each haplotype statistic into non-overlapping windows of 100 kb pairs. We identified regions with the longest haplotypes reaching significance thresholds of *α* = 0.01. For XP-EHH, we compared Mesoamericans to East Asians to look specifically for haplotypes present in Mesoamerican populations that arose after their split from Asian populations. For iHS, the windows were binned by the number of SNPs for quantile estimation of percentile using Selscan's norm function. iHS scores were not computed for a MAF < 0.05. Windows with fewer than 10 SNPs were dropped from analysis, which included the loss of 18,779 SNPs in the iHS dataset and 24,245 in the XP-EHH dataset. Only windows in the 1% tail of bin distribution were considered. We found 316 XP-EHH windows and 203 iHS windows significant at the 1% level.

LSBL was paired the XP-EHH and iHS haplotype tests. Only windows that fell within the 1% tail for both paired tests were considered as candidates for positive selection. Here, we find that only 57 iHS plus LSBL windows and 100 XP-EHH and iHS windows passed this threshold.

We tested how admixture and imputing from our own dataset affected our allele frequency analyses by using the program Ohana with our merged dataset consisting of the Mesoamerican cohort plus CEU, YRI, CHB + JPT. This program analyzes population structure and outputs admixture-corrected allele frequencies (Cheng et al. 2022). We conducted a supervised population structure analysis with *K* = 4, and then used the output matrix of admixture-corrected allele frequencies to calculate pairwise *F*_st_ and LSBL for each SNP for the same populations used above. These results validated our original LSBL findings for our candidate regions under selection.

As our Allele frequency test was unaffected by the imputation, we determined that our imputed dataset had been minimally affected by cryptic population substructure by homogenizing certain regions. To test this, we re-ran iHS and XP-EHH and normalized for the entire dataset with admixed individuals at the locus of interest, if the region was still in the 1% further analysis was not necessary. If the region dropped out of the 1% significance, a separate run was conducted for a subset of individuals who were unadmixed at the window. To determine who was unadmixed at each specific locus, we used a 4-population admixture model between Indigenous Americans (SGDP), Africans (YRI), Han Chinese (CHB), and Europeans (CEU). We considered each of the window assignments, pulling out only those individuals for which an Indigenous American ancestry was reported in both the paternally and maternally inherited chromosomal haplotypes.

### Annotation of Regions

For both the selection scan and introgression analysis, windows were annotated for genes using the bedmaps option from BEDOPS tools (Neph et al. 2012).

### HLA Allele Calls

HLA allele calls were imputed using the multi-ethnic HLA (version 1.0 2021) reference panel as part of the Michigan Imputation Server HLA-TAPAS pipeline (Das et al. 2016; Luo et al. 2021). QC criteria used were MAF > 0.01 and rsq > 0.3 (Pistis et al. 2015). To confirm accuracy of the HLA calls, we downloaded the high coverage alignment files for the HGDP and SGDP individuals from Central/Southern Mexico through the European Bioinformatics Institute (EMBL-EBI) endpoint on GLOBUS (Mallick et al. 2016; Bergström et al. 2020). These individuals included 21 Maya, 3 Mixe, 2 Mixtec, and 2 Zapotec. HLA calls were generated using HLA-LA, which takes the alignment files and realigns them to a graph genome (Dilthey et al. 2019). One Zapotec sample failed to run and was removed from our analysis. Each individual's HLA-DPA1 and DPB1 calls with coverage and probability are available in the supplementals.

### Estimating Haplotype Ages

To estimate the haplotype age, we used a method that employs EHH scores and assumes a starlike phylogeny to assess the age of decay from a core marker (Reich and Goldstein 1999; Sabeti et al. 2002b; Voight et al. 2006). We calculated the EHH statistic for our core SNPs using Selscan v1.3.0 until EHH decay reached 0.05 (Hardwick et al. 2014; Szpiech and Hernandez 2014). Given that EHH≈Pr(Homozygosity), or the probability of homozygosity, we can use the following equation:  Pr(Homozygosity)=e^−2*RG*^, *R* = haplotype length in M (morgans), *G* = generation time marker (Reich and Goldstein 1999; Sabeti et al. 2002b; Voight et al. 2006). This equation can be rearranged and reduced to a simple slope-intercept form (*y* = *mx* + *b*) through the origin (*b* = 0) by taking the natural log of the EHH values and doubling the distance in morgans from the core, which gives us the equation −ln(EHH) = *G* × 2*R*. This rearrangement of the data allows us to determine the generation time (slope of equation) since the haplotype arose using a linear regression (Hardwick et al. 2014). We calculated the regression coefficients using a linear model through the origin and generated a 95% confidence in *R* (Team 2013). The raw output, with residuals and coefficients, is available in the supplementals. To calculate a rough estimate of age in years, we assumed a 25-year generation time.

## Supplementary Material

evad015_Supplementary_Data

## Data Availability

Data will be shared on the author's GitHub page: https://github.com/obedaram/Mesoamerican-Data.
